# Di-μ-chlorido-bis­{aqua­chlorido[3-ethyl-4-phenyl-5-(2-pyrid­yl)-4*H*-1,2,4-triazole-κ^2^ *N* ^1^,*N* ^5^]manganese(II)}

**DOI:** 10.1107/S1600536808044103

**Published:** 2009-01-08

**Authors:** Zuoxiang Wang, Ixaoning Gong, Chunyi Liu, Xiaoming Zhang

**Affiliations:** a Ordered Matter Science Research Center, Southeast University, Nanjing 210096, People’s Republic of China; b Jiangsu Institute of Nuclear Medicine, Wuxi 214063, People’s Republic of China

## Abstract

In the centrosymmetric dinuclear title compound, [Mn_2_Cl_4_(C_15_H_14_N_4_)_2_(H_2_O)_2_], the Mn^II^ atom is coordinated by an *N*,*N*′-bidentate ligand, a water mol­ecule, a terminal chloride ion and two bridging chloride ions in a distorted MnN_2_OCl_3_ octa­hedral geometry. The Mn⋯Mn separation is 3.6563 (9) Å. In the crystal structure, O—H⋯N and O—H⋯Cl hydrogen bonds help to establish the packing.

## Related literature

For background, see: Klingele *et al.* (2005 ▶), Kume *et al.* (2006 ▶).
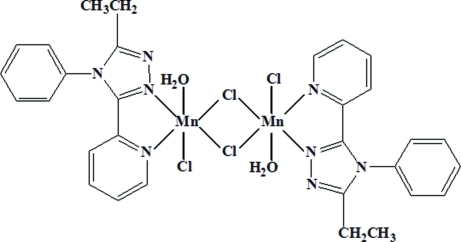


## Experimental

### 

#### Crystal data

[Mn_2_Cl_4_(C_15_H_14_N_4_)_2_(H_2_O)_2_]
*M* *_r_* = 788.32
Monoclinic, 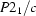

*a* = 9.9369 (15) Å
*b* = 8.9369 (13) Å
*c* = 19.642 (3) Å
β = 103.323 (2)°
*V* = 1697.3 (4) Å^3^
*Z* = 2
Mo *K*α radiation
μ = 1.10 mm^−1^
*T* = 293 (2) K
0.32 × 0.26 × 0.24 mm

#### Data collection

Bruker SMART APEX CCD diffractometer
Absorption correction: multi-scan (*SADABS*; Bruker, 2000 ▶) *T*_min_ = 0.72, *T*_max_ = 0.77
8811 measured reflections
3329 independent reflections
2364 reflections with *I* > 2σ(*I*)
*R*_int_ = 0.045

#### Refinement

*R*[*F* ^2^ > 2σ(*F* ^2^)] = 0.053
*wR*(*F* ^2^) = 0.105
*S* = 1.02
3329 reflections
209 parameters
H-atom parameters constrained
Δρ_max_ = 0.35 e Å^−3^
Δρ_min_ = −0.44 e Å^−3^

### 

Data collection: *SMART* (Bruker, 2000 ▶); cell refinement: *SAINT* (Bruker, 2000 ▶); data reduction: *SAINT*; program(s) used to solve structure: *SHELXTL* (Sheldrick, 2008 ▶); program(s) used to refine structure: *SHELXTL*; molecular graphics: *SHELXTL*; software used to prepare material for publication: *SHELXTL*.

## Supplementary Material

Crystal structure: contains datablocks I, global. DOI: 10.1107/S1600536808044103/hb2883sup1.cif

Structure factors: contains datablocks I. DOI: 10.1107/S1600536808044103/hb2883Isup2.hkl

Additional supplementary materials:  crystallographic information; 3D view; checkCIF report

## Figures and Tables

**Table:** 

Mn1—O1	2.273 (2)
Mn1—N2	2.280 (3)
Mn1—N1	2.344 (3)
Mn1—Cl2	2.4544 (11)
Mn1—Cl1	2.5252 (11)
Mn1—Cl1^i^	2.5387 (11)

**Table:** 

Mn1—Cl1—Mn1^i^	92.45 (4)

Symmetry code: (i) 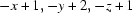.

**Table 2:** Hydrogen-bond geometry (Å, °)

*D*—H⋯*A*	*D*—H	H⋯*A*	*D*⋯*A*	*D*—H⋯*A*
O1—H1*A*⋯Cl2^ii^	0.85	2.28	3.122 (3)	170
O1—H1*C*⋯N3^ii^	0.85	2.12	2.875 (4)	148

Symmetry code: (ii) 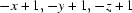.

**Fig. 1.**
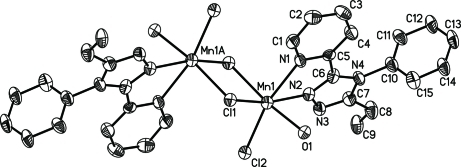
The molecular structure of (I) with Displacement ellipsoids shown at the 30% probability level and H atoms omitted for clarity. Mn1A and the unlabelled atoms are generated by the symmetry operation (1–x, 2–y, 1–z).

## supplementary crystallographic information

### Comment

The 1,2,4-triazole ring can act as a bidentate ligand in coordination chemistry (e.g. Klingele *et al.*, 2005; Kume *et al.* 2006).  We report here the synthesis and crystal structure analysis of the title compound, (I).

The structure of (I) is shown in Fig.1. The title compound is a centrosymmetric dinuclear maganese(II) complex bridged by two chloride ions (Table 1). The dihedral angle between the triazole and pyridine rings is 9.42 (24)°, and that between the triazole and benzene rings is 80.53 (12)°. In the crystal, O—H···N and O—H···Cl hydrogen bonds (Table 2) help to establish the packing.

### Experimental

To a warm solution of 0.501 g of 3-ethyl-4-phenyl-5-(2-pyridyl)-1,2,4-triazole (2.0 mmol) in 10 ml ethanol, 0.792 g of manganese(II) chloride tetrahydrate (4.0 mmol) in 10 ml water was added. The filtrate was left to stand at room temperature for several days, and pale yellow blocks of (I) were collected.

### Refinement

The H atoms were gemoetrically placed (C—H = 0.93–0.97Å, O—H = 0.85Å) and refined as riding with U_iso_(H) = 1.2U_eq_(carrier) or 1.5U_eq_(methyl C).

### Crystal data

**Table:**

[Mn_2_Cl_4_(C_15_H_14_N_4_)_2_(H_2_O)_2_]	*F*(000) = 804
*M**_r_* = 788.32	*D*_x_ = 1.542 Mg m^−^^3^
Monoclinic, *P*2_1_/*c*	Mo *K*α radiation, λ = 0.71073 Å
Hall symbol: -P 2ybc	Cell parameters from 4766 reflections
*a* = 9.9369 (15) Å	θ = 2.5–28.0°
*b* = 8.9369 (13) Å	µ = 1.10 mm^−^^1^
*c* = 19.642 (3) Å	*T* = 293 K
β = 103.323 (2)°	Block, pale yellow
*V* = 1697.3 (4) Å^3^	0.32 × 0.26 × 0.24 mm
*Z* = 2	

### Data collection

**Table:**

Bruker SMART APEX CCD diffractometer	3329 independent reflections
Radiation source: sealed tube	2364 reflections with *I* > 2σ(*I*)
graphite	*R*_int_ = 0.045
ω scans	θ_max_ = 26.0°, θ_min_ = 2.1°
Absorption correction: multi-scan (*SADABS*; Bruker, 2000)	*h* = −12→8
*T*_min_ = 0.72, *T*_max_ = 0.77	*k* = −11→10
8811 measured reflections	*l* = −24→24

### Refinement

**Table:**

Refinement on *F*^2^	Primary atom site location: structure-invariant direct methods
Least-squares matrix: full	Secondary atom site location: difference Fourier map
*R*[*F*^2^ > 2σ(*F*^2^)] = 0.053	Hydrogen site location: inferred from neighbouring sites
*wR*(*F*^2^) = 0.105	H-atom parameters constrained
*S* = 1.02	*w* = 1/[σ^2^(*F*_o_^2^) + (0.04*P*)^2^ + 0.95*P*] where *P* = (*F*_o_^2^ + 2*F*_c_^2^)/3
3329 reflections	(Δ/σ)_max_ < 0.001
209 parameters	Δρ_max_ = 0.35 e Å^−^^3^
0 restraints	Δρ_min_ = −0.44 e Å^−^^3^

### Special details

**Table:**

Geometry. All e.s.d.'s (except the e.s.d. in the dihedral angle between two l.s. planes) are estimated using the full covariance matrix. The cell e.s.d.'s are taken into account individually in the estimation of e.s.d.'s in distances, angles and torsion angles; correlations between e.s.d.'s in cell parameters are only used when they are defined by crystal symmetry. An approximate (isotropic) treatment of cell e.s.d.'s is used for estimating e.s.d.'s involving l.s. planes.
Refinement. Refinement of *F*^2^ against ALL reflections. The weighted *R*-factor *wR* and goodness of fit *S* are based on *F*^2^, conventional *R*-factors *R* are based on *F*, with *F* set to zero for negative *F*^2^. The threshold expression of *F*^2^ > σ(*F*^2^) is used only for calculating *R*-factors(gt) *etc*. and is not relevant to the choice of reflections for refinement. *R*-factors based on *F*^2^ are statistically about twice as large as those based on *F*, and *R*- factors based on ALL data will be even larger.

### Fractional atomic coordinates and isotropic or equivalent isotropic displacement parameters (Å^2^)

**Table:**

	*x*	*y*	*z*	*U*_iso_*/*U*_eq_	
Mn1	0.52358 (6)	0.80195 (6)	0.52295 (3)	0.03231 (16)	
Cl1	0.32636 (10)	0.98690 (10)	0.50448 (4)	0.0327 (2)	
Cl2	0.46719 (10)	0.68828 (10)	0.40606 (4)	0.0340 (2)	
C1	0.5362 (4)	0.9652 (4)	0.67503 (19)	0.0356 (9)	
H1	0.4723	1.0295	0.6477	0.043*	
C2	0.5739 (4)	0.9897 (5)	0.7457 (2)	0.0416 (10)	
H2	0.5393	1.0715	0.7654	0.050*	
C3	0.6643 (4)	0.8909 (5)	0.78734 (19)	0.0404 (10)	
H3	0.6896	0.9038	0.8356	0.049*	
C4	0.7167 (4)	0.7719 (4)	0.75560 (18)	0.0365 (9)	
H4	0.7757	0.7025	0.7825	0.044*	
C5	0.6801 (3)	0.7581 (4)	0.68393 (18)	0.0263 (7)	
C6	0.7326 (4)	0.6459 (4)	0.64299 (19)	0.0324 (8)	
C7	0.8480 (4)	0.4695 (4)	0.60588 (18)	0.0335 (8)	
C8	0.9328 (5)	0.3335 (5)	0.60667 (19)	0.0438 (10)	
H8A	1.0122	0.3415	0.6459	0.053*	
H8B	0.8786	0.2488	0.6159	0.053*	
C9	0.9834 (4)	0.2988 (5)	0.5451 (2)	0.0422 (10)	
H9A	0.9074	0.2977	0.5047	0.063*	
H9B	1.0272	0.2024	0.5506	0.063*	
H9C	1.0492	0.3734	0.5390	0.063*	
C10	0.8737 (4)	0.4760 (4)	0.73627 (18)	0.0368 (9)	
C11	1.0054 (4)	0.5134 (5)	0.77226 (18)	0.0390 (9)	
H11	1.0620	0.5727	0.7517	0.047*	
C12	1.0518 (4)	0.4594 (5)	0.84098 (19)	0.0397 (10)	
H12	1.1397	0.4835	0.8670	0.048*	
C13	0.9654 (4)	0.3702 (5)	0.86918 (19)	0.0420 (10)	
H13	0.9960	0.3335	0.9144	0.050*	
C14	0.8336 (5)	0.3345 (5)	0.83126 (19)	0.0421 (10)	
H14	0.7758	0.2764	0.8517	0.051*	
C15	0.7878 (5)	0.3844 (5)	0.7637 (2)	0.0443 (10)	
H15	0.7012	0.3570	0.7372	0.053*	
N1	0.5877 (3)	0.8514 (3)	0.64330 (14)	0.0303 (7)	
N2	0.7055 (3)	0.6526 (3)	0.57301 (15)	0.0322 (7)	
N3	0.7784 (3)	0.5400 (3)	0.55068 (15)	0.0329 (7)	
N4	0.8223 (3)	0.5325 (3)	0.66508 (14)	0.0313 (7)	
O1	0.3944 (3)	0.6254 (3)	0.56175 (12)	0.0321 (6)	
H1A	0.4420	0.5461	0.5721	0.039*	
H1C	0.3228	0.6062	0.5299	0.039*	

### Atomic displacement parameters (Å^2^)

**Table:**

	*U*^11^	*U*^22^	*U*^33^	*U*^12^	*U*^13^	*U*^23^
Mn1	0.0361 (3)	0.0276 (3)	0.0307 (3)	0.0000 (2)	0.0025 (2)	0.0027 (2)
Cl1	0.0357 (5)	0.0278 (4)	0.0319 (4)	−0.0004 (4)	0.0022 (4)	0.0028 (3)
Cl2	0.0396 (5)	0.0281 (5)	0.0320 (4)	0.0000 (4)	0.0033 (4)	0.0023 (3)
C1	0.044 (2)	0.0233 (18)	0.039 (2)	0.0110 (16)	0.0080 (17)	−0.0013 (16)
C2	0.043 (2)	0.040 (2)	0.044 (2)	0.0019 (18)	0.0136 (19)	−0.0123 (18)
C3	0.040 (2)	0.054 (3)	0.0277 (18)	0.002 (2)	0.0079 (17)	−0.0088 (18)
C4	0.039 (2)	0.039 (2)	0.0292 (18)	0.0055 (18)	0.0043 (16)	0.0020 (16)
C5	0.0177 (15)	0.0252 (17)	0.0346 (17)	−0.0063 (13)	0.0027 (14)	0.0008 (14)
C6	0.037 (2)	0.0271 (18)	0.0341 (19)	0.0037 (16)	0.0110 (17)	0.0057 (15)
C7	0.039 (2)	0.0322 (19)	0.0292 (18)	0.0090 (17)	0.0079 (16)	−0.0013 (15)
C8	0.061 (3)	0.041 (2)	0.031 (2)	0.021 (2)	0.0134 (19)	0.0053 (17)
C9	0.040 (2)	0.045 (2)	0.042 (2)	0.0178 (19)	0.0108 (18)	0.0159 (19)
C10	0.045 (2)	0.035 (2)	0.0272 (17)	0.0145 (18)	0.0007 (17)	0.0003 (16)
C11	0.047 (2)	0.043 (2)	0.0253 (17)	0.0156 (19)	0.0036 (18)	−0.0038 (16)
C12	0.041 (2)	0.045 (2)	0.0313 (19)	0.0245 (19)	0.0045 (18)	0.0087 (17)
C13	0.047 (3)	0.047 (2)	0.0293 (19)	0.024 (2)	0.0044 (18)	0.0118 (17)
C14	0.049 (3)	0.043 (2)	0.0313 (19)	0.0236 (19)	0.0022 (18)	0.0072 (16)
C15	0.042 (2)	0.052 (3)	0.038 (2)	0.007 (2)	0.0074 (19)	0.0102 (19)
N1	0.0344 (17)	0.0319 (16)	0.0232 (14)	0.0032 (13)	0.0036 (13)	0.0050 (12)
N2	0.0368 (18)	0.0262 (16)	0.0313 (16)	0.0005 (13)	0.0029 (14)	0.0041 (12)
N3	0.0374 (18)	0.0287 (16)	0.0303 (15)	0.0047 (14)	0.0027 (13)	0.0028 (12)
N4	0.0355 (17)	0.0337 (16)	0.0224 (14)	0.0055 (14)	0.0019 (13)	0.0034 (12)
O1	0.0367 (14)	0.0260 (13)	0.0330 (13)	−0.0022 (11)	0.0066 (11)	0.0033 (10)

### Geometric parameters (Å, °)

**Table:**

Mn1—O1	2.273 (2)	C7—C8	1.477 (5)
Mn1—N2	2.280 (3)	C8—C9	1.447 (5)
Mn1—N1	2.344 (3)	C8—H8A	0.9700
Mn1—Cl2	2.4544 (11)	C8—H8B	0.9700
Mn1—Cl1	2.5252 (11)	C9—H9A	0.9600
Mn1—Cl1^i^	2.5387 (11)	C9—H9B	0.9600
Cl1—Mn1^i^	2.5387 (11)	C9—H9C	0.9600
C1—N1	1.353 (5)	C10—C11	1.377 (6)
C1—C2	1.369 (5)	C10—C15	1.379 (6)
C1—H1	0.9300	C10—N4	1.463 (4)
C2—C3	1.385 (6)	C11—C12	1.407 (5)
C2—H2	0.9300	C11—H11	0.9300
C3—C4	1.393 (5)	C12—C13	1.378 (6)
C3—H3	0.9300	C12—H12	0.9300
C4—C5	1.376 (5)	C13—C14	1.387 (6)
C4—H4	0.9300	C13—H13	0.9300
C5—N1	1.356 (4)	C14—C15	1.374 (5)
C5—C6	1.455 (5)	C14—H14	0.9300
C6—N2	1.340 (5)	C15—H15	0.9300
C6—N4	1.354 (5)	N2—N3	1.370 (4)
C7—N3	1.306 (4)	O1—H1A	0.8500
C7—N4	1.368 (4)	O1—H1C	0.8500
			
O1—Mn1—N2	84.37 (10)	C9—C8—H8B	107.7
O1—Mn1—N1	80.58 (10)	C7—C8—H8B	107.7
N2—Mn1—N1	70.80 (10)	H8A—C8—H8B	107.1
O1—Mn1—Cl2	90.09 (7)	C8—C9—H9A	109.5
N2—Mn1—Cl2	98.54 (8)	C8—C9—H9B	109.5
N1—Mn1—Cl2	166.35 (8)	H9A—C9—H9B	109.5
O1—Mn1—Cl1	91.32 (7)	C8—C9—H9C	109.5
N2—Mn1—Cl1	163.18 (8)	H9A—C9—H9C	109.5
N1—Mn1—Cl1	92.47 (8)	H9B—C9—H9C	109.5
Cl2—Mn1—Cl1	97.71 (3)	C11—C10—C15	122.9 (4)
O1—Mn1—Cl1^i^	172.57 (7)	C11—C10—N4	119.2 (4)
N2—Mn1—Cl1^i^	94.62 (8)	C15—C10—N4	117.8 (4)
N1—Mn1—Cl1^i^	92.13 (8)	C10—C11—C12	118.2 (4)
Cl2—Mn1—Cl1^i^	97.34 (4)	C10—C11—H11	120.9
Cl1—Mn1—Cl1^i^	87.55 (4)	C12—C11—H11	120.9
Mn1—Cl1—Mn1^i^	92.45 (4)	C13—C12—C11	119.1 (4)
N1—C1—C2	122.9 (3)	C13—C12—H12	120.4
N1—C1—H1	118.6	C11—C12—H12	120.4
C2—C1—H1	118.6	C12—C13—C14	121.1 (4)
C1—C2—C3	119.1 (4)	C12—C13—H13	119.4
C1—C2—H2	120.5	C14—C13—H13	119.4
C3—C2—H2	120.5	C15—C14—C13	120.3 (4)
C2—C3—C4	118.7 (3)	C15—C14—H14	119.8
C2—C3—H3	120.6	C13—C14—H14	119.8
C4—C3—H3	120.6	C14—C15—C10	118.3 (4)
C5—C4—C3	119.2 (4)	C14—C15—H15	120.9
C5—C4—H4	120.4	C10—C15—H15	120.9
C3—C4—H4	120.4	C1—N1—C5	117.9 (3)
N1—C5—C4	122.0 (3)	C1—N1—Mn1	124.2 (2)
N1—C5—C6	112.3 (3)	C5—N1—Mn1	117.9 (2)
C4—C5—C6	125.7 (3)	C6—N2—N3	107.5 (3)
N2—C6—N4	108.9 (3)	C6—N2—Mn1	114.8 (2)
N2—C6—C5	121.6 (3)	N3—N2—Mn1	135.8 (2)
N4—C6—C5	129.2 (3)	C7—N3—N2	107.8 (3)
N3—C7—N4	109.9 (3)	C6—N4—C7	105.9 (3)
N3—C7—C8	126.7 (3)	C6—N4—C10	128.6 (3)
N4—C7—C8	123.2 (3)	C7—N4—C10	125.3 (3)
C9—C8—C7	118.3 (3)	Mn1—O1—H1A	109.5
C9—C8—H8A	107.7	Mn1—O1—H1C	109.5
C7—C8—H8A	107.7	H1A—O1—H1C	109.5

Symmetry codes: (i) −*x*+1, −*y*+2, −*z*+1.

### Hydrogen-bond geometry (Å, °)

**Table:**

*D*—H···*A*	*D*—H	H···*A*	*D*···*A*	*D*—H···*A*
O1—H1A···Cl2^ii^	0.85	2.28	3.122 (3)	170
O1—H1C···N3^ii^	0.85	2.12	2.875 (4)	148

Symmetry codes: (ii) −*x*+1, −*y*+1, −*z*+1.
